# Exploring the formation of public acceptability of biodiversity offsetting in Finland

**DOI:** 10.1111/cobi.70169

**Published:** 2025-10-26

**Authors:** Tuija Seppälä, Kaisa Raatikainen, Liisa Varumo, Iikka Oinonen, Heidi Lehtiniemi, Riikka Paloniemi, Suvi Huttunen

**Affiliations:** ^1^ Finnish Environment Institute Helsinki Finland

**Keywords:** biodiversity offsetting, concern, hopefulness, public acceptability, aceptación pública, biodiversidad, compensación, optimismo, preocupación

## Abstract

Public acceptance of nature conservation instruments is critical for their effective and fair implementation. Understanding conservation governance as a rational activity aligns with the view that citizens base their judgments of conservation instruments on a critical evaluation of the anticipated ecological, economic, and social impacts. However, although citizens generally consider various conservation instruments acceptable, their awareness and knowledge about the instruments are often limited, suggesting that the judgments may also be influenced by factors other than knowledge. We explored acceptability of voluntary biodiversity offsetting in Finland that was written into law in 2023. We hypothesized that public acceptability of the instrument hinges partially on the level of concern for biodiversity loss and of hopefulness that biodiversity loss can be halted and that level of acceptability is justified through rational arguments regarding the instrument's impacts. We tested our hypotheses empirically with an online survey of a representative sample of Finnish citizens (*n* = 1993). Biodiversity offsetting was largely unknown to the public; yet, it was generally judged to be highly acceptable. The supportive argument was that biodiversity offsetting promotes resolution of conservation conflicts, and the opposing arguments were that it restricts land use and leads to degradation of nature values. Hopefulness amplified the positive effect of concern for biodiversity on acceptability of offsetting (β = 0.072). Acceptability was positively related to the argument that biodiversity offsetting results in resolution of conservation conflicts (β = 0.424) and negatively related to the claims that it results in restriction of land use (β = −0.213) and destruction of nature values (β = −0.195). Our results broaden theoretical understanding of the public acceptance of conservation policies.

## Abstract (es)

Análisis de la aceptación pública de la compensación de la biodiversidad en Finlandia

**Resumen:**

La aceptación pública de los instrumentos de conservación de la naturaleza es fundamental para su aplicación eficaz y justa. Entender la gobernanza de la conservación como una actividad racional concuerda con la opinión de que los ciudadanos basan sus juicios sobre los instrumentos de conservación en una evaluación crítica de los impactos ecológicos, económicos y sociales previstos. Sin embargo, aunque los ciudadanos suelen aceptar diversos instrumentos de conservación, su conciencia y conocimiento sobre los mismos suele ser limitada, lo que sugiere que los juicios también pueden verse influidos por factores distintos del conocimiento. Exploramos cuánto se acepta la compensación voluntaria de la biodiversidad en Finlandia que se incorporó a la legislación en 2023. Planteamos la hipótesis de que la aceptación pública del instrumento depende en parte del nivel de preocupación por la pérdida de biodiversidad y de la esperanza de que esta pérdida pueda detenerse, y que el nivel de aceptación se justifica mediante argumentos racionales sobre los impactos del instrumento. Probamos nuestra hipótesis empíricamente con una encuesta en línea realizada a una muestra representativa de ciudadanos finlandeses (*n*=1993). La compensación de la biodiversidad era en gran medida desconocida por el público, pero en general se consideraba muy aceptable. El argumento a favor era que la compensación de la biodiversidad promueve la resolución de los conflictos relacionados con la conservación; los argumentos en contra eran que restringe el uso de la tierra y conduce a la degradación de los valores naturales. El optimismo amplificó el efecto positivo de la preocupación por la biodiversidad en la aceptación de la compensación (β = 0.072). La aceptación se relacionó positivamente con el argumento de que la compensación de la biodiversidad da lugar a la resolución de conflictos relacionados con la conservación (β = 0.424) y se relacionó negativamente con las afirmaciones de que da lugar a la restricción del uso del suelo (β = ‐0.213) y a la destrucción de los valores naturales (β = ‐0.195). Nuestros resultados amplían la comprensión teórica de la aceptación pública de las políticas de conservación.

## INTRODUCTION

Human activities threaten biodiversity. Global estimates of terrestrial land affected by land use range from 75% (Mu et al., 2022; Venter et al., 2016) to 95% (Kennedy et al., 2019). Deterioration of nature is the most severe where human influence is the greatest, including settlements and agricultural land (Kennedy et al., 2019; Venter et al., 2016). Yet, the overall human impact on biodiversity is even more pervasive when the constituents of the biosphere are analyzed (Elhacham et al., 2020; Greenspoon et al., 2023). Thus, new conservation instruments are needed urgently to halt biodiversity loss and safeguard nature as human activities expand into new areas (Bull & Strange, 2018; Calvet et al., 2015; Díaz et al., 2019). However, to be effectively adopted and implemented, conservation policy instruments require public acceptance (Blicharska et al., 2016).

Public acceptance of conservation instruments has received significant attention from researchers and policy makers (e.g., Ejelöv & Nilsson, 2020; Jones et al., 2022). In acceptance studies, researchers typically conceptualize citizens as rational decision makers who base their judgments on all available information and a critical evaluation of the anticipated ecological, economic, and social impacts of the instruments. Numerous studies show that these evaluations matter (e.g., Bergquist et al., 2022; Drews & van den Bergh, 2016; Ejelöv & Nilsson, 2020; Huber et al., 2020; Huijts et al., 2012; Jones et al., 2022), but the studies are based on cross‐sectional designs, and alternative interpretations of the findings have not been tested thoroughly (Ejelöv & Nilsson, 2020). Public awareness, knowledge, and understanding of conservation instruments are generally limited, but even poorly known and understood instruments are widely seen as acceptable by the public (e.g., Cole et al., 2022; Gaspers et al., 2024; O'Connor et al., 2021; Manson et al., 2021). This suggests that judgments may also be based on issues other than knowledge.

We explored how public acceptability is formed in the adoption of voluntary biodiversity offsetting under new legislation in Finland (BBOP, 2012; Maron et al., 2016; Moilanen & Kotiaho, 2021). We explored policy attitudes through the concept of acceptability rather than acceptance because acceptability is more applicable to emerging policies. In contrast, acceptance is a more relevant concept for existing policies (Kyselá et al., 2019). Globally, biodiversity offsetting is not a new conservation instrument, having been utilized in dozens of countries since the 1970s (Calvet et al., 2015). In Finland, biodiversity offsetting was only recently introduced as a voluntary conservation instrument in the updated Nature Conservation Act in 2023. The instrument is widely supported by Finnish experts and stakeholders (Lehtiniemi et al., 2023; Karlsson & Karhunmaa, 2024; Varumo et al., 2023), but there is no prior knowledge on the overall level of public awareness of or the acceptability of biodiversity offsetting in Finland or on attitudes toward the details of the offsetting measure among citizens.

We also explored how public acceptability of a new conservation measure may be formed. We propose that overall judgment of the acceptability of a poorly known conservation instrument may be formed intuitively based on the perceived importance of the problem (concerns for biodiversity loss) and the belief that positive change is possible (the hopefulness of halting biodiversity loss) and that the stated level of overall acceptability is justified through rational arguments regarding the impacts of the instrument. The role of intuitive judgments and heuristic thinking—concepts from human behavioral sciences (e.g., Gilovich et al., 2002; Kahneman, 2011; Zajonc, 1980)—has been recognized in the environmental policy acceptability literature (e.g., Ejelöv & Nilsson, 2020; Perlaviciute & Steg, 2014). However, empirical studies are limited particularly regarding the relationship between acceptability and the evaluation of policy impacts. Further, understanding of the interactive effects of environmental concern and hope on the public acceptability of conservation policies is limited. Our empirical study is based on a survey conducted among citizens shortly after the Nature Conservation Act in Finland was updated. Our study broadens the theoretical understanding of policy acceptance, and we suggest that there is a need for integrative frameworks that consider psychological determinants of attitudes toward new conservation initiatives. This would provide a more comprehensive understanding of the factors influencing the overall acceptance of conservation.

### Acceptability of biodiversity offsetting among the public

Societies are responding to the ecological deterioration of their environments in broader terms than merely banning most destructive human activities. Conservation policies and governance have evolved from state‐led conservation efforts toward multilevel, polycentric, and networked forms of governance, in which the roles of collaborative arrangements, market actors, and the creation of markets and economic value for biodiversity are increasing (Bonneuil, 2015; Bouwma et al., 2018; Lemos & Agrawal, 2006). This development is visible globally in diverse contexts from China to the United States and European Union (Chiavacci & Pindilli, 2020; Hermoso et al., 2022; Mu et al., 2022); the emergence and spread of biodiversity offsetting is but one example.

Biodiversity offsetting refers to a scheme that compensates for the destruction of biodiversity in one place by restoring or protecting nature in another, aiming to prevent overall degradation of ecological values (Moilanen & Kotiaho, 2021). Unlike traditional conservation efforts, which focus on preserving existing natural areas, biodiversity offsetting allows for the loss of biodiversity in certain locations so long as equivalent or greater biodiversity gains are provided elsewhere, with the aim of no net loss or a net gain of biodiversity (Bonneuil, 2015; Bull et al., 2013; Calvet et al., 2015). This approach integrates conservation objectives with developmental needs, leveraging market‐based instruments as a means of achieving both goals (Bonneuil, 2015). In recent decades, many countries have introduced biodiversity offsetting schemes (Bull & Strange, 2018; Calvet et al., 2015). These schemes, sometimes termed *ecological compensation*, *compensatory mitigation*, or *mitigation banking*, vary considerably in their scope, ecological metrics, governmental organization, and practical implementation (Droste et al., 2022; Hrabanski, 2015). Yet, ensuring the effectiveness of offsetting and verifiability of no‐net‐loss or net‐gain aims remains uncertain (Maron et al., 2025).

Biodiversity offsetting contains several aspects that make public acceptability important. Although biodiversity offsetting is fundamentally about protecting biodiversity, the protection occurs in socioecological contexts and has implications for both development and offsetting areas (Apostolopoulou & Adams, 2017; Tupala et al., 2022). The local impacts can be either negative or positive and may affect the livelihoods of local people, opportunities for recreation in the local landscape, or the continuity of local identity (Bidaud et al., 2017; Griffiths et al., 2019; Maron et al., 2016; Tupala et al., 2022). Therefore, the acceptance and success of biodiversity offsetting at local level depend on balancing these ecological, economic, and social considerations to meet the diverse needs and values of affected communities. Beyond local acceptance, biodiversity offsetting raises a societal question about the way conservation is conducted and a more philosophical question about the substitutability of nature (Apostolopoulou, 2020; Ives & Bekessy, 2015; Tupala et al., 2022). Although the local impacts can only be grasped at a general level from a survey, we focused on the societal impacts.

Research on citizens’ opinions of biodiversity offsetting at a principal level remains scarce. However, existing studies suggest that the biodiversity offsetting instrument has a relatively high level of public acceptability (Burton et al., 2017; Cole et al., 2022; Scholte et al., 2016). For example, Cole et al. (2022) conducted a representative survey in the Skåne region of Sweden and found that 64% of respondents considered the principle of biodiversity offsetting a good idea, whereas 11% thought it was a bad idea. Only 24% of respondents reported having previously heard about the instrument (Cole et al., 2022). However, research on the overall acceptability of biodiversity offsetting has generally been atheoretical, focusing primarily on the role of socioeconomic factors. For example, Cole et al. (2022) reported that women, younger people, highly educated individuals, medium‐ and high‐income groups, parents, and dog owners were more likely to have a positive attitude toward the offsetting instrument.

### Formation of public acceptability in the context of new policy measures

Public support of nature conservation instruments is critical for their effective and fair implementation. In particular, voluntary conservation instruments may face reluctance from companies and public actors if residents and customers perceive them as lacking acceptance and legitimacy (Varumo et al., 2023). The implementation and expansion of these instruments often depend on the willingness of residents and private landowners to collaborate with local decision makers (Calvet et al., 2019). The process of selecting policy instruments is considered by some sociopolitical rather than purely rational (Cho & Moon, 2019). This means, for instance, that public opinion significantly drives environmental policies (Anderson et al., 2017; Van Eeden et al., 2020). Decision makers frequently hesitate to implement policies without strong constituent support because disapproval can result in poor policy uptake (Takacs, 2020). Therefore, it is crucial to understand the factors influencing public policy attitudes like acceptability.

*Attitude* can be defined in various ways. According to the classic definition, attitude is “a psychological tendency that is expressed by evaluating a particular entity with some degree of favor or disfavor” (Eagly & Chaiken, 1993, p. 1). Other researchers emphasize the situationally constructed nature of attitude, defining it as an evaluative judgment that can be formed when needed (Schwarz, 2007; Schwarz & Bohner, 2001). The psychological function of attitudes is to inform individuals about adaptive behavior in specific situations. Thus, people can construct attitudes toward unfamiliar objects when asked based on whatever information seems relevant and available at the time (Schwarz, 2007; Schwarz & Bohner, 2001). Relevant information may be related to the features of the object, affective reactions within the respondent, or the respondent's own behavior regarding the object (Schwarz & Bohner, 2001).

While forming a judgment of a new conservation instrument, individuals may draw their stance from their more general environmental attitude, which indicates how an individual feels about nature based on their values (cf., Homer & Kahle, 1988; Rokeach, 1968; Schwartz, 1992). Environmental concern is often thought to reflect this kind of general attitude toward nature (Bamberg, 2003; Dunlap & Jones, 2002; Schultz, 2001; Stern & Dietz, 1994). Different underlying values can result in different types of concern. Schultz (2001) distinguishes between egoistic, altruistic, and biospheric environmental concern. Egoistic concern focuses on the consequences for oneself, altruistic concern on the consequences for other people, and biospheric concern on the consequences for nature, reflecting differences in underlying personal values (Gifford & Nilsson, 2014; Schultz, 2001).

Environmental concern is positively associated with interest in conservation (e.g., Hansla et al., 2013; McCarty & Shrum, 1994; Stern & Dietz, 1994) and support for conservation policies (e.g., Borg et al., 2024; Manson et al., 2021). However, a specific conservation instrument may not resonate with all types of environmental concern. Biodiversity offsetting as an instrument, based on the idea that a loss of biodiversity in one place can be offset by human interventions in another (Moilanen & Kotiaho, 2021), violates the intrinsic value of nature—the idea that nature in a specific place and time is unique and valuable (Karlsson & Edvardsson Björnberg, 2021). Thus, biodiversity offsetting may appear less acceptable to individuals who are more concerned about the impacts of biodiversity loss on nature than to those who are more concerned about enabling development activities (Damiens et al., 2021; Lehtiniemi et al., 2023). However, biodiversity offsetting, as such, does not recognize the social values attached to nature, making it potentially less acceptable to those more concerned about the impacts of biodiversity loss on human well‐being (cf. Karlsson & Edvardsson Björnberg, 2021; Tupala et al., 2022).

The rational approach to public policy attitudes assumes that individuals critically evaluate policy impacts before forming their attitudes. Conversely, the intuitive approach suggests that political preferences determine the arguments people find compelling (e.g., Kahneman, 2011). In line with this, we propose that individuals find arguments related to the impacts of biodiversity offsetting compelling when these arguments align with their concern‐informed attitudes. By aligning with attitude‐congruent arguments and rejecting attitude‐inconsistent arguments, individuals may also rationally justify their stated overall judgments of the policy instrument and maintain cognitive consistency (e.g., Gawronski & Brannon, 2019). Consequently, acceptability ratings might be the cause, rather than the result, of the evaluation of policy‐specific impacts.

We propose that concern about biodiversity loss is positively related to the overall acceptability of biodiversity offsetting. This acceptability, in turn, is positively associated with positive claims and negatively associated with negative claims about the impacts of biodiversity offsetting. Because concern about biodiversity loss reflects a more general attitude toward nature, it may also directly influence agreement and disagreement with specific claims. We consolidated these propositions into one mediation hypothesis: The overall acceptability of biodiversity offsetting partially mediates the effects of concern about biodiversity loss on agreement with claims about the impacts of biodiversity offsetting (H1).

### Moderating role of hopefulness

Although environmental concern can motivate individuals and communities toward environmental protection, it can also lead to paralyzing effects on proenvironmental attitudes and behaviors (e.g., Innocenti et al., 2023; Qin et al., 2024). Awareness of environmental degradation has contributed to the rise of dystopian scenarios of societal change, fueling anxiety, apathy, and other paralyzing sentiments in societies (Bennett et al., 2016). Belief in an irreversible, black future can become a self‐fulfilling prophecy because people tend to behave according to their beliefs and expectations about the future (Merton, 1948; Ostrom et al., 2002). To counteract these potentially paralyzing effects, people need hope that positive change is achievable (Bennett et al., 2016; Brophy et al., 2023; Dean & Wilson, 2023; Fredrickson, 2001; O'Neill & Nicholson‐Cole, 2009; Ojala, 2007).

Although hope can serve as a psychological force that builds resilience and helps people cope with crises (Fredrickson, 2001), hope without concern can lead to denial of environmental problems, unrealistic optimism about business as usual, or exclusive reliance on technological innovations to solve issues (Ojala, 2023; Pleeging et al., 2021). Thus, there is a delicate balance between concern and hope. Hope can mitigate the effects of concern, and concern ensures that hope remains critical and constructive (Ojala, 2023; Pleeging et al., 2021).

Thus, we complemented H1 with 2 additional hypotheses. The hopefulness of halting biodiversity loss moderates the effect of concern on overall acceptability, strengthening the positive effect when the level of hopefulness is higher relative to when it is lower (H2). And, the hopefulness of halting biodiversity loss moderates the residual direct effects of concern on agreement with diverse biodiversity offsetting claims. Specifically, a higher level of hopefulness strengthens agreement with positive claims, whereas a lower level of hopefulness strengthens agreement with negative claims (H3).

The conceptual model is in Appendix S1. The model suggests that overall acceptability mediates the effect of concern on justifications (agreement and rejection of specific claims about the impacts of biodiversity offsetting) and that these indirect effects are conditional on the level of hopefulness. In addition, the model suggests that the residual, direct effects of hopefulness on justifications are conditional on the level of hopefulness.

## METHODS

The study was part of a larger survey. Data were gathered through an online survey during 1 week in April–May 2023. The survey was administered by a specialist market research company, Kantar Public. The company collected the data through their established probability‐based online panel of the Finnish general public. Although online panels are a popular and efficient method for data collection, they present certain limitations that may affect the representativeness and quality of the data (e.g., Callegaro et al., 2014). For instance, probability‐based internet panels often have low recruitment participation rates (Hays et al., 2015). An active sampling approach was used to ensure that the final sample was representative of the population by age, gender, and region. Respondents were at least 18 years of age and represented residents of mainland Finland. The language of the survey was Finnish, which meant that non‐Finnish speakers were not invited to participate in the survey. Altogether, 2058 responses were received. Externally motivated respondents of online panels may engage in a variety of less‐than‐optimal strategies that can lead to a variety of undesirable responses (Hays et al., 2015). The responses of 65 participants were removed due to their fast response time, indicating a lack of attention to the questions. We used a conservative 2‐s per question limit (Huang et al., 2012). Thus, the final sample included 1993 respondents. The survey protocol followed the guidelines of the Finnish National Board on Research Integrity (TENK). According to TENK's guidelines, certain designs in human sciences require an ethical review (TENK, 2019, p. 19). None of these designs were applicable to our study; hence, the study did not undergo an ethical review.

### Measures

Biodiversity is a well‐known concept among adults in Finland (Ranta & Ahtinen, 2022); thus, we expected that our respondents would be familiar with it. Concern for biodiversity loss was measured with one item: “Are you concerned about biodiversity loss in Finland?” Respondents rated their concern on a 5‐point scale: 1, *not at all concerned*; 2, *a bit concerned*; 3, *quite concerned*; 4, *concerned*; 5, *extremely concerned*. There was also an “I do not know” option for all the questions in this study. A single‐item measure was used to capture the general level of concern for biodiversity loss and to limit respondent burden in a large survey.

Accordingly, hopefulness of halting biodiversity loss was measured with one item: “Are you hopeful that the loss of biodiversity in Finland will be halted?” Respondents rated their hopefulness on a 5‐point scale: 1, *not at all hopeful*; 2, *a bit hopeful*; 3, *quite hopeful*; 4, *hopeful*; 5, *very hopeful*.

We provided a short contextual description of biodiversity offsetting (Appendix S2) before asking respondents’ level of awareness of the instrument. Based on a Eurobarometer 2018 questionnaire (European Commission, 2019), we asked: “Have you heard of the concept ‘biodiversity offsetting’ before?” Participants responded on a 3‐point scale: 1, *I have heard about it, and I know what it means*; 2, *I have heard about it, but I do not know what it means*; 3, *I have not heard about it before*. We scored awareness in the responses as 1, *high level*; 2, *medium level*; and 3, *low level*.

A short description of biodiversity offsetting (Appendix S3) was provided for the respondents before asking about the overall acceptability of the measure with one question: “Do you consider this kind of biodiversity offsetting an acceptable practice?” The respondents rated acceptability on a 5‐point scale: 1, *not at all acceptable*; 2, *not very acceptable*; 3, *quite acceptable*; 4, *acceptable*; 5, *totally acceptable*.

Justifications for the overall acceptability of biodiversity offsetting were explored through 15 biodiversity offsetting–related claims, which are presented in the Results section. The claims were based on arguments presented by various stakeholders during the preparation for updating the Nature Conservation Act in Finland (Lehtiniemi et al., 2023). Respondents were asked to express their opinion on each of the claims on a 5‐point scale: 1, *totally disagree*; 2, *disagree*; 3, *neither disagree nor agree*; 4, *agree*; 5, *totally agree*.

### Analytical strategy

Before hypothesis testing, we conducted an explorative factor analysis for the 15 biodiversity offsetting claims with the Psych package in R 4.1.0 to see whether the items could be grouped in any meaningful way. For these items, item‐level missingness ranged from 5% to 20%; therefore, we used a full information maximum likelihood (FIML) estimation to extract factors as recommended (e.g., Newman, 2014) and an oblique rotation (Promax with Kaiser Normalization). We used a mix of objective criteria, including Kaiser criterion, parallel analysis, and the elbow point method, and considered the interpretability of the solution to decide the final number of factors (Lloret‐Segura et al., 2014). We calculated sum variables of the items accordingly. In further analyses, we used these sum variables as end variables and considered them representative of the different types of justifications for the overall acceptability of the biodiversity offsetting instrument. Mediation and moderated mediation analyses were conducted with the Lavaan package in R. We used FIML estimation with a bootstrapping approach to test total, direct, and indirect effects (Shrout & Bolger, 2002). Missing data ranged from 2% to 15% depending on the variable. All the predictor variables were standardized by dividing by the grand mean before the analysis. Interactions were probed at 1 SD below and above the mean levels of the moderator. We also tested whether the mediation and moderated mediation models similarly applied to the different levels of biodiversity offsetting awareness. For that purpose, we added awareness as a grouping variable to the model and estimated an unconstrained model in which all regression coefficients were allowed to differ between the 3 levels of awareness (low, medium, high) and a constrained model where all regression coefficients were equal across the 3 levels of awareness. We compared the unconstrained model and the constrained model with a chi‐square difference test.

## RESULTS

### Descriptive results of concern, hopefulness, awareness, and acceptability

**FIGURE 1:**
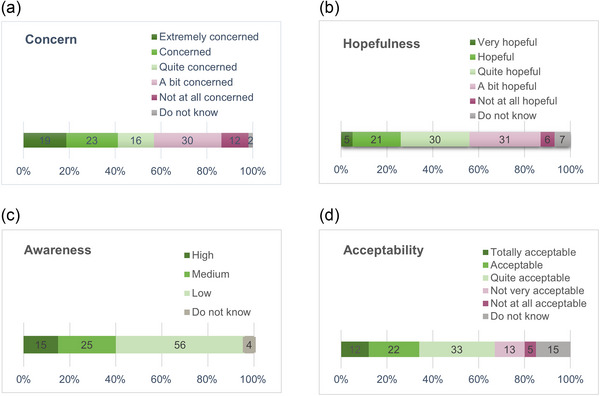
Frequencies of (a) concern for biodiversity loss, (b) hopefulness of halting biodiversity loss, (c) awareness of the biodiversity offsetting instrument, and (d) acceptability of biodiversity offsetting among respondents (*n* = 1993) to a survey of the general public in Finland. Total percentages may exceed 100% due to rounding.

Most of the respondents reported being concerned about biodiversity loss in Finland (Figure 1a); only 12% expressed that they were not at all concerned. Respondents were moderately hopeful that biodiversity loss in Finland will be halted (Figure 1b). As expected, biodiversity offsetting was a relatively unknown instrument among the respondents (Figure 1c). Only 15% reported that they had heard about biodiversity offsetting and knew what it meant. Nonetheless, biodiversity offsetting was generally considered an acceptable instrument; 67% of the respondents considered it at least “quite acceptable” (Figure 1d).

### Grouping biodiversity offsetting claims into justifications

During the exploratory factor analysis, it appeared that one claim, “biodiversity offsetting will lower the threshold for degrading biodiversity,” had a cross loading over 0.30 on 2 factors, so we removed it from the analysis (Howard, 2016). Kaiser–Mayer–Olkin (KMO) test value was 0.81, suggesting that the data were suitable for factor analysis. The scree plot of eigenvalues indicated a sharp drop after 2 factors, suggesting that 2 factors should be retained. However, parallel analysis suggested retaining 5 factors. Therefore, we considered the final solution to lie between these 2 extremes. A qualitative comparison between 3‐ and 4‐factor solutions revealed that the 3‐factor solution was more parsimonious. Consequently, the final solution included 3 factors that explained 49% of the total variance (Table 1).

**TABLE 1:** Results from a factor analysis of biodiversity offsetting (BO) claims from a survey of the Finnish general public (*n* = 1993).

Biodiversity offsetting claim factor and item^a^	Factor loading^b^		
	1	2	3
1. Resolution of conservation conflicts (α = 0.85)			
BO will increase flexibility of conservation.	0.89^*^	0.10	0.02
BO will promote the preservation of biodiversity.	0.83^*^	0.08	−0.10
BO will help to consider the goals of conservation and other land use simultaneously.	0.70^*^	−0.02	−0.10
It will be better to offset than to let biodiversity be destroyed.	0.59^*^	−0.15	0.10
BO will increase the acceptability of development projects.	0.57^*^	0.12	−0.06
BO will improve the reputation of the actor causing the loss of biodiversity.	0.57^*^	0.07	0.06
BO will bring more money to conservation.	0.52^*^	−0.04	−0.03
BO will create new income opportunities for landowners.	0.47^*^	−0.17	0.11
2. Restriction of land use (α = 0.82)			
BO will significantly restrict the rights of landowners.	0.02	0.93^*^	0.04
BO will unreasonably increase the costs of development projects.	0.05	0.79^*^	0.02
3. Degradation of nature values (α = 0.60)			
Biodiversity destroyed in one place cannot be offset elsewhere.	−0.03	−0.01	0.59^*^
BO will result in greenwashing.	−0.11	0.11	0.54^*^
BO will impair recreational opportunities in residential green spaces.	−0.04	−0.01	0.54^*^
The value of nature should not be measured in money.	0.10	−0.01	0.43^*^

^a^ Cronbach alpha in parentheses.

^b^ Asterisk: primary factor loading.

Eight items, loading mainly on factor 1, emphasized the positive ecological, social, and economic impacts that biodiversity offsetting can result in. We named this factor *resolution of conservation conflicts* because its essence lies in the idea that biodiversity offsetting can resolve the conflicts between economic development and conservation (Bull & Brownlie, 2017).

Items focusing on the risks of biodiversity offsetting loaded mainly on factors 2 and 3. Factor 2 captured 2 claims, suggesting constrained impacts on land use and expressing concern that biodiversity offsetting will hinder economic activities. We named this factor *restriction of land use*. Four items loading mainly on factor 3 included claims about the irreplaceability of nature, the risk of greenwashing, the loss of local nature, and the commodification of nature. We named this factor *degradation of nature values*.

### Mediating effect of acceptability on the relationship between concern and justifications

The correlations between the variables provided preliminary support for the hypothesis that the relationship between concern and different types of claims is partially mediated by overall acceptability (Table 2).

**TABLE 2:** Descriptive statistics and correlations for study variables in mediation and moderated mediation models examining the acceptability of biodiversity offsetting (*n* = 1993).^a^

Variable^b^	*M*	SD	Factor 1	Factor 2	Factor 3	Concern	Hope
Factor 1	3.42	0.71					
Factor 2	2.96	1.02	−0.24^***^				
Factor 3	33.68	0.74	−0.10^***^	−0.05			
Concern	3.07	1.33	0.22^***^	−0.46^***^	0.26^***^		
Hope	2.88	1.02	0.02	0.17^***^	−0.14^***^		
Acceptability	3.28	1.06	0.61^***^	−0.34^***^	−0.17^***^	0.33^***^	−0.06^**^

^a^ Significance: **p < 0.01; ***p < 0.001.

^b^ Factors defined in Table 1.

**FIGURE 2:**
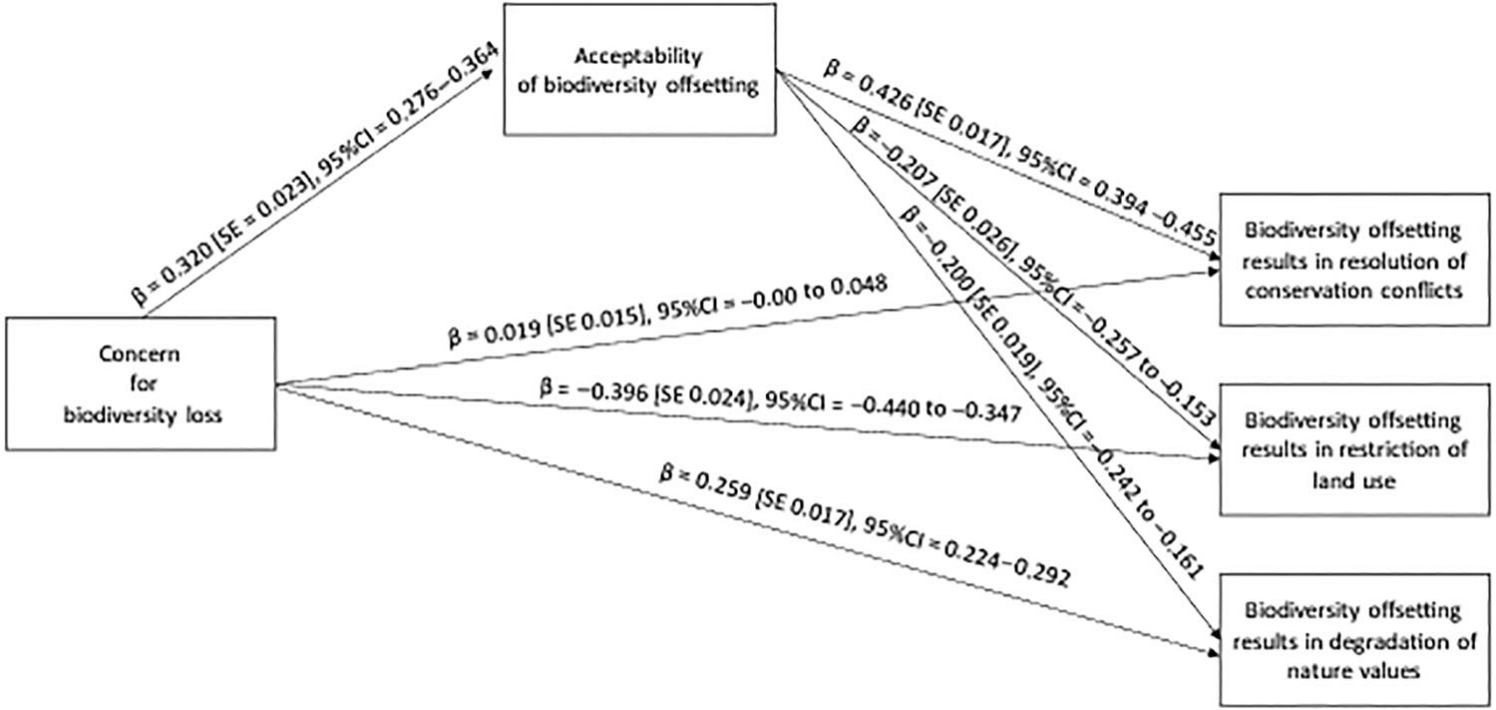
Standardized direct effects in a mediation model that tests if the overall acceptability of biodiversity offsetting partially mediates the effects of concern about biodiversity loss on agreement with claims about the impacts of biodiversity offsetting.

As we assumed, concern was positively related to the overall acceptability of biodiversity offsetting, which in turn was positively related to the claim that biodiversity offsetting results in resolution of conservation conflicts and negatively related to the claims that biodiversity offsetting results in restriction of land use and degradation of nature values (Figure 2).

The mediation analysis revealed that concern had an indirect positive effect, based on overall acceptability, on the claim that biodiversity offsetting results in resolution of conservation conflicts (Table 3). However, it had no statistically significant direct effect (Figure 2), suggesting that overall acceptability fully mediated the relationship. Concern had indirect negative effects, based on overall acceptability, on the claims that biodiversity offsetting results in restriction of land use and degradation of nature values, whereas its direct effects on these claims remained statistically significant, suggesting that mediation was partial. The total effect of concern on the claim that biodiversity offsetting results in restriction of land use was negative, whereas there was a positive total effect on the claims that biodiversity offsetting results in resolution of conservation conflicts and degradation of nature values. These findings supported hypothesis H1.

**TABLE 3:** Indirect and total effects of concern for biodiversity loss on the 3 biodiversity offsetting claims (resolution of conservation conflicts, restriction of land use, and degradation of nature values) as determined in a survey of the general public in Finland.

Indirect effect	β	SE	95% CI
concern → acceptability → resolution of conservation conflicts	0.136	0.012	0.114 to 0.159
concern → acceptability → restriction of land use	−0.066	0.010	−0.088 to −0.047
concern → acceptability → degradation of nature values	−0.064	0.008	−0.081 to −0.050
**Total effect**			
concern → resolution of conservation conflicts	0.155	0.017	0.122 to 0.188
concern → restriction of land use	−0.462	0.022	−0.509 to −0.419
concern → degradation of nature values	0.195	0.017	0.162 to 0.227

The constrained model (all regression coefficients were equal across the 3 levels of awareness) did not differ significantly from the saturated unconstrained model (Δχ^2^ = 18.32, df = 14, *p* = 0.341), suggesting that the parameters were equal at all levels of prior awareness.

### Moderating effects of hopefulness

**FIGURE 3:**
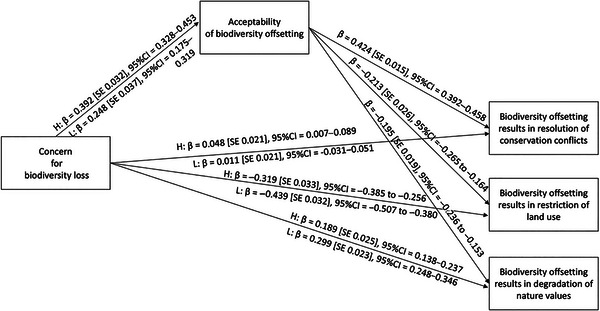
Standardized conditional direct effects and direct effects in a moderated mediation model used to test if the hopefulness of halting biodiversity loss moderates the effect of concern on overall acceptability, strengthening the positive effect when the level of hopefulness is higher relative to when it is lower, and if the hopefulness of halting biodiversity loss moderates the residual direct effects of concern on agreement with diverse biodiversity offsetting claims. H, high level of hopefulness of halting biodiversity loss (+1 SD); L, low level of hopefulness of halting biodiversity loss (−1 SD).

Concern and hope had a statistically significant interaction effect on overall acceptability (β = 0.072 [SE 0.025], 95% CI = 0.0023–0.121). Specifically, the positive effect of concern on overall acceptability was stronger when the level of hopefulness was high (Figure 3). Although the effect was weaker when hopefulness was lower, it remained positive and statistically significant. Overall acceptability, in turn, had statistically significant effects on the claims that biodiversity offsetting results in resolution of conservation conflicts, restriction of land use, and degradation of nature values.

The conditional indirect effect of concern on the claim that biodiversity offsetting results in resolution of conservation conflicts based on overall acceptability was stronger when the level of hope was high than when it was low (Table 4). Indirect effects remained positive and statistically significant in both conditions. Likewise, the conditional indirect effect of concern on the claim that biodiversity offsetting results in restriction of land use based on overall acceptability was stronger when the level of hope was high than when it was low. Indirect effects remained negative and statistically significant in both conditions. Finally, the conditional indirect effect of concern on the claim that biodiversity offsetting results in degradation of nature values was stronger when the level of hope was high than when it was low. Indirect effects remained negative and statistically significant in both conditions. Thus, our findings supported H2.

**TABLE 4:** Conditional indirect effects of concern for biodiversity loss on the 3 biodiversity offsetting claims (resolution of conservation conflicts, restriction of land use, and degradation of nature values) as determined in a survey of the general public in Finland.

Conditional indirect effect	β	SE	95% CI
concern × high hope → acceptability → resolution of conservation conflicts	0.166	0.016	0.137 to 0.196
concern × low hope → acceptability → resolution of conservation conflicts	0.105	0.016	0.072 to 0.136
concern × high hope → acceptability → restriction of land use	−0.083	0.012	−0.107 to −0.061
concern × low hope → acceptability → restriction of land use	−0.053	0.011	−0.074 to −0.033
concern × high hope → acceptability → degradation of nature values	−0.076	0.010	−0.094 to −0.058
concern × low hope → acceptability → degradation of nature values	−0.048	0.008	−0.065 to −0.033

Hope moderated the residual direct effects of concern on claims that biodiversity offsetting results in restriction of land use (β = 0.060 [SE 0.022], 95% CI 0.016–0.107) and degradation of nature values (β = −0.055 [0.016], 95% CI −0.088 to −0.026). The direct conditional residual effect of concern on the claim that biodiversity offsetting results in restriction of land use was stronger when the level of hope was low than when it was high. Direct effects remained negative and statistically significant in both conditions (Figure 3). Finally, the direct conditional residual effect of concern on the claim that biodiversity offsetting results in degradation of nature values was stronger when the level of hope was low than when it was high. Direct effects remained positive and statistically significant in both conditions. Thus, our findings supported hypothesis H3.

In our comparison of the groups with different levels of prior awareness, the constrained model did not differ significantly from the saturated unconstrained model (Δχ^2^ = 49.51, df = 30, *p* = 0.059), suggesting that the parameters were equal at all levels of prior awareness.

## DISCUSSION

We found that biodiversity offsetting was relatively unknown among the Finnish public but was still widely judged as acceptable. This aligns with previous research indicating that the public generally holds an overall positive attitude toward various conservation instruments despite having limited understanding and knowledge of them (e.g., Cole et al., 2022; Gaspers et al., 2024; Kokkoris et al., 2023; Manson et al., 2021; O'Connor et al., 2021). This evidence suggests that people can form an attitude toward a conservation instrument even with very limited prior knowledge about it.

Based on attitude research, we proposed that the public's specific attitudes toward new conservation instruments are influenced by their perceived importance of the problem, reflected in their concern for biodiversity loss, and their belief in the possibility of positive change, reflected in their hopefulness of halting biodiversity loss. We discovered that concern and hopefulness had a positive interaction effect on the overall acceptability of biodiversity offsetting. Our results demonstrated that hopefulness amplified the positive effect of environmental concern on conservation policy acceptability, and even low levels of hope did not negate the positive impact of concern on support for biodiversity conservation. This is a new empirical contribution to the previous research on the predictive relationship between environmental concern and positive attitudes toward various environmental policies among citizens (e.g., Bergquist et al., 2022; Borg et al., 2024; Bouman et al., 2020; Goldberg et al., 2021; Hansla et al., 2013; Smith & Leiserowitz, 2014). However, our results did not support the idea that concern would have a paralyzing effect without hope (Bennett et al., 2016). For example, we did not find that the effect of concern on policy acceptability would be negative when the level of hope is low. This might indicate that, although the majority of Finns report being concerned for the loss of biodiversity (Ranta & Ahtinen, 2022), dystopian views of environmental change are still scarce among the Finnish public.

Our findings also indicated that biodiversity offsetting did not address all types of concern. Specifically, we found that overall acceptability fully mediated the positive relationship between concern for biodiversity loss and the tendency to find the argument that biodiversity offsetting results in resolution of conservation conflicts compelling. This suggests that the instrument particularly appeals to those who value biodiversity conservation without compromising other land‐use goals. These sentiments align with the premises of the biodiversity offsetting instrument (Moilanen & Kotiaho, 2021). Conversely, our results showed that environmental concern had a negative indirect effect, via overall acceptability, on the tendency to find the argument that biodiversity offsetting leads to degradation of nature values compelling, whereas the residual direct effect was positive. This implies that the instrument does not resonate with those who value local nature as unique. These sentiments are in line with the ethical and social challenges commonly associated with the biodiversity offsetting instrument (Ives & Bekessy, 2015; Karlsson & Edvardsson Björnberg, 2021; Maron et al., 2016). Additionally, a low level of concern was associated with more negative judgments of biodiversity offsetting and a tendency to find the argument that it results in restriction of land use compelling. These sentiments may reflect a negative stance on nature conservation in general and may not be specifically related to the characteristics of the biodiversity offsetting instrument (e.g., Ejelöv & Nilsson, 2020).

The general positive attitude toward biodiversity offsetting among the public aligns with the overall supportive stance of various stakeholders in Finland, such as environmental civil society organizations and representatives of public governance (Lehtiniemi et al., 2023; Karlsson & Karhunmaa, 2024; Varumo et al., 2023). This broad positive stance, both among the public and expert stakeholders, likely reflects the widespread concern for biodiversity loss in Finland. National surveys indicate that public awareness of biodiversity issues has recently increased, partly due to the topic's visibility in the media and citizens being increasingly concerned about the state of nature in Finland (e.g., Ranta & Ahtinen, 2022). Another potential explanation is that biodiversity offsetting is a new instrument in Finland, with limited or no practical experience among the involved parties (Pietilä et al., 2024). Prior research suggests that although biodiversity offsetting is generally rated as highly acceptable in principle, this acceptability is not without qualifications (Burton et al., 2017; Cole et al., 2022; Lehtiniemi et al., 2023). We did not study public preferences, but potential qualifications might widen the gap between public acceptability in principal and opposition during public hearings or landowners’ willingness to provide land for biodiversity offsetting (Bell et al., 2005).

Our results add to the understanding that values play a key role in shaping public responses to conservation. Although value change is often proposed as a long‐term solution to conservation challenges (e.g., Manfredo et al., 2016), values tend to be stable and slow to evolve (e.g., Manfredo et al., 2017), and shifts in values do not necessarily translate into desired behavioral outcomes (e.g., Kyle & Landon, 2023). More effective and immediate strategies may lie in targeting attitudes, social norms, and specific behaviors within their contextual settings (e.g., Manfredo et al., 2017). Although public attitudes toward biodiversity offsetting as a market‐based instrument were generally favorable, a closer examination revealed a range of perspectives and concerns. These insights are valuable for policy makers and conservation practitioners because they highlight the importance of addressing public expectations and apprehensions when designing and implementing offsetting schemes and broader conservation policy frameworks.

Our study has several limitations. First, much of the prior research on attitudes toward conservation instruments relies on cross‐sectional designs, which allow for alternative interpretations of results that have not been thoroughly tested (Ejelöv & Nilsson, 2020). Although we provided an examination of an alternative interpretation, it remains tentative due to its cross‐sectional nature. Future studies could employ designs that facilitate the comparison between the knowledge‐based model and the concern‐based model (e.g., Lammer & Gollwitzer, 2017).

Second, when respondents are asked to express their specific attitudes toward unfamiliar issues in surveys, their attitudes are often constructed on the spot and are heavily influenced by how the information is presented, framed, and contextualized (Tourangeau et al., 2000). In our study, for example, the way biodiversity offsetting was introduced or defined before asking respondents about their overall attitude may have significantly shaped their responses. Specifically, introducing biodiversity offsetting by emphasizing ongoing biodiversity degradation may have created a positive contextual framing for the new conservation instrument. This framing effect may also be present in other studies reporting relatively positive overall attitudes toward biodiversity offsetting among the public (e.g., Cole et al., 2022). Moving forward, we suggest that survey studies on attitudes toward conservation policies pay closer attention to the potential influence of presentation, framing, and contextual effects. Addressing these factors is essential to obtain more reliable estimates of public acceptance and ensuring that the results genuinely reflect public attitudes rather than artifacts of survey design.

Third, our findings suggest that respondents expressed concern for biodiversity loss for various reasons; however, the single‐item measure of concern we used did not allow for a detailed exploration of this issue. Although multidimensional scales exist to measure both concern (e.g., Cruz & Manata, 2020) and hope (e.g., Pleeging, 2022), we opted for single‐item measures. Single‐item measures have several advantages, especially in large‐scale surveys, such as saving respondents’ time and being more satisfying for them (e.g., Allen et al., 2022). However, multi‐item scales often outperform single‐item scales in reliability and validity (e.g., Franzen & Mader, 2021; Pleeging, 2022). Given the declining public interest in participating in survey studies, it is crucial to develop efficient ways to measure psychological constructs (Allen et al., 2022).

According to our results, in general, Finns considered the legally mandated voluntary biodiversity offsetting to be an acceptable conservation instrument. However, citizens justified the acceptability of biodiversity offsetting from different perspectives, and these justifications were partly contradictory. Because biodiversity offsetting as a conservation instrument was scarcely known, it is reasonable to assume that acceptability and its justifications were largely based on respondents’ more general attitudes toward nature conservation. From this perspective, it can be stated that Finland's compensation model is a compromise that reconciles the diverse interests of citizens but may potentially lead to trade‐offs concerning the biodiversity benefits achieved through biodiversity offsetting. One key trade‐off relates to the voluntary nature of the instrument, which creates a situation in which the conservation outcome depends on willingness to compensate. Because not all land‐use development projects are obliged to compensate for their negative impacts on biodiversity, the overall benefits for nature may be reduced and the no‐net‐loss principle not achieved nationally. Respondents recognized these trade‐off risks despite the conservation instrument being new to them. This supports the conclusion that citizens assess the acceptability of new policy measures more broadly than based on knowledge—that is, based on how the policy measures support citizens’ values and how citizens envision the future in relation to the policy issue at hand.

## Supporting information



Supporting information

Supporting information

Supporting information
