# Supplementary figures and images for: Exploring the formation of public acceptability of biodiversity offsetting in Finland
Source: Conserv Biol. 2025 Oct 26;40(2):e70169. doi: 10.1111/cobi.70169 (PMC13036305; doi:10.1111/cobi.70169)

Appendix S1

Conceptual model of attitude formation.


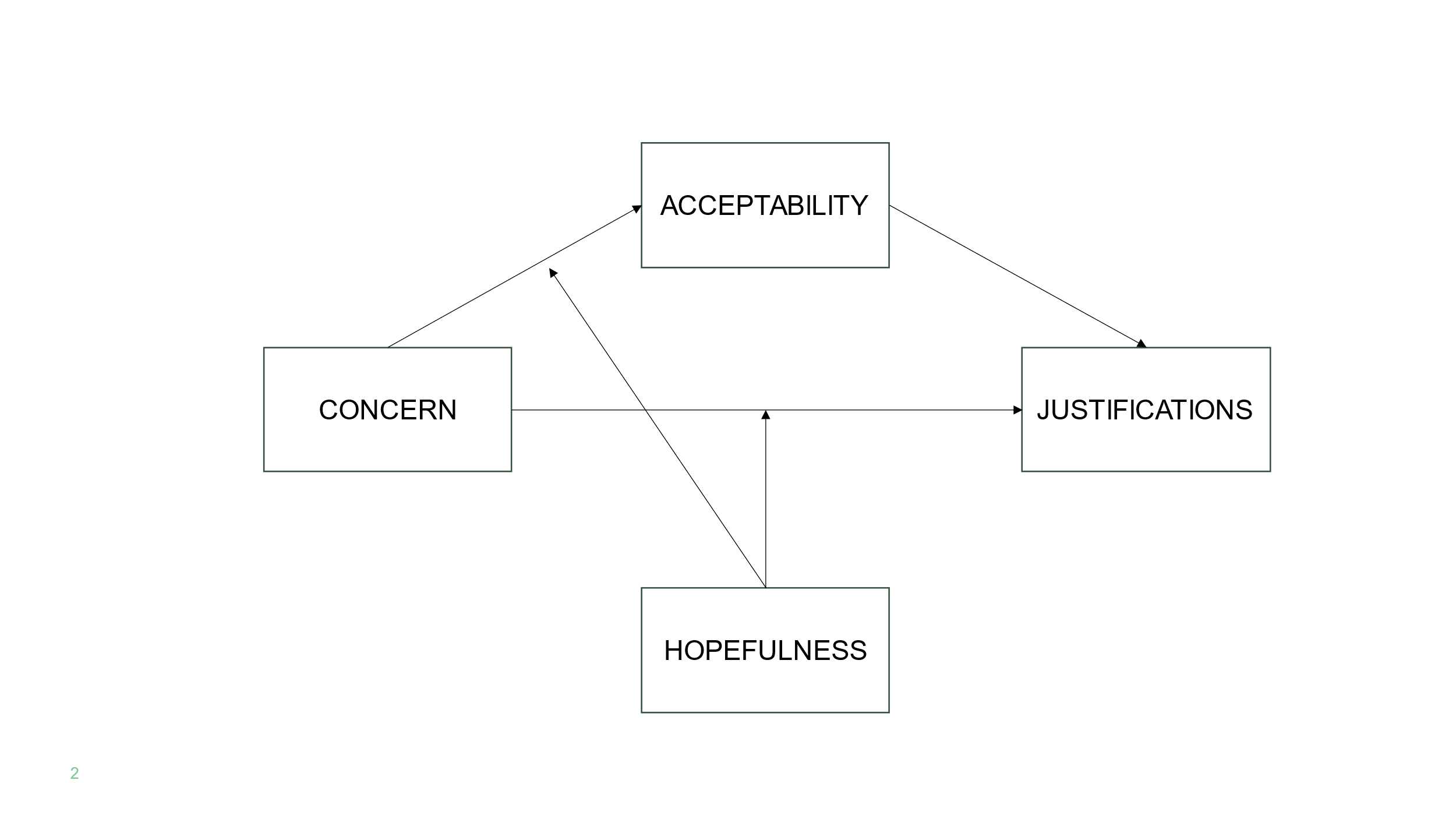

Supplement: Supplementary file 1 — Supporting information [file COBI-40-e70169-s001.docx]
