# Supplementary material for: Exploring the formation of public acceptability of biodiversity offsetting in Finland
Source: Conserv Biol. 2025 Oct 26;40(2):e70169. doi: 10.1111/cobi.70169 (PMC13036305; doi:10.1111/cobi.70169)
Supplement: Supplementary file 2 — Supporting information [file COBI-40-e70169-s002.docx]

Appendix S2

A short description of the context provided for the participants in the survey before asking their level of awareness of biodiversity offsetting instrument.

“Finland is committee to halting biodiversity loss both internationally and nationally, but despite this, the biodiversity of Finland’s nature continues to decline. The new Nature Conservation Act includes voluntary biodiversity offsetting as one means to help ensure the preservation of biodiversity. Biodiversity offsetting is different from carbon offsetting, for example, in the context of air travel.”
