# Supplementary material for: Exploring the formation of public acceptability of biodiversity offsetting in Finland
Source: Conserv Biol. 2025 Oct 26;40(2):e70169. doi: 10.1111/cobi.70169 (PMC13036305; doi:10.1111/cobi.70169)
Supplement: Supplementary file 3 — Supporting information [file COBI-40-e70169-s003.docx]

Appendix S3

A short description of biodiversity offsetting provided for the participants in the survey before asking its overall acceptability.

"Biodiversity is decreasing, meaning habitats are disappearing and deteriorating in quality as humans use land and natural resources for their purposes. When it is not technically possible or economically feasible to avoid or mitigate the harm caused to nature at the site of the impact, efforts can be made to prevent biodiversity loss through biodiversity offsetting, for example.

Biodiversity offsetting refers to a procedure where the party causing the environmental harm improves the condition of similar nature elsewhere, so that the overall state of nature does not deteriorate. In practice, for example, a company can buy or produce new ecological values equivalent to the environmental harm it has caused. Landowners can produce ecological values by protecting, restoring, or rehabilitating natural sites.

The goal of biodiversity offsetting is to fully compensate for the environmental harm caused, taking into account the risks of failure in nature improvement measures. The adequacy of the compensation in relation to the environmental harm caused is ensured by standardized calculation rules. Using standardized calculation rules, the required amount of biodiversity offsetting can be calculated consistently across different projects."
